# Histone modification and non-coding RNAs in skin aging: emerging therapeutic avenues

**DOI:** 10.1515/biol-2025-1222

**Published:** 2025-12-30

**Authors:** Yuchang Wang, Kang Wang, Qi Zhang, Yukun Liu

**Affiliations:** Division of Trauma Surgery, Emergency Surgery & Surgical Critical, Tongji Hospital, Tongji Medical College, Huazhong University of Science and Technology, Wuhan, 430030, China; Trauma Center, Tongji Hospital, Tongji Medical College, Huazhong University of Science and Technology, Wuhan, 430030, China; Department of Emergency and Critical Care Medicine, Tongji Hospital, Tongji Medical College, Huazhong University of Science and Technology, Wuhan, 430030, China; Sino-German Research Institute of Disaster Medicine, Huazhong University of Science and Technology, Wuhan, 430030, China; Department of Plastic and Aesthetic Surgery, Tongji Hospital, Tongji Medical College, Huazhong University of Science and Technology, Wuhan, 430030, China

**Keywords:** skin aging, epigenetic modification, therapeutic targets, non-coding RNAs, histone modification

## Abstract

Skin aging is a lifelong process that begins after birth and is characterized by a decline in morphology and function. This deterioration is associated with the appearance of wrinkles, increased laxity, and fragility, which may increase the risk of age-related skin diseases. Despite extensive research in the past few decades, the underlying mechanism of skin aging remains unknown. Epigenetic modifications, which refer to potentially heritable alterations without changes in DNA sequence, including DNA methylation, histone modification, and non-coding RNAs (ncRNAs), have been implicated in regulating skin aging through intrinsic and extrinsic interactions. In this review, we summarize the available clinical and experimental studies to elucidate the mechanisms of epigenetic regulation in skin aging, with a focus on DNA methylation, histone modification, and ncRNAs (such as miRNA, lncRNA, and circRNA). Epigenetic inheritance has been shown to regulate the senescence and collagen synthesis of skin cells, which can interfere with skin aging. Therefore, this review highlights the molecular mechanisms underlying skin aging and potential therapeutic targets for intervention, while also identifying directions for reversing skin aging.

## 1 Introduction

Skin is the largest organ of the human body, covering an area of approximately 1.8m2 and comprising three stratified regions: the epidermis, dermis, and hypodermis. The epidermis contains spinous, granular, and corneous layers, and a basal layer rich in keratinocytes and epidermal stem cells, which are responsible for skin renewal [1], 2]. The dermis consists of fibroblasts that maintain the extracellular matrix (ECM) homeostasis by synthesizing and secreting collagen, fibronectin, elastin, and matrix metalloproteinases [3]. The hypodermis contains adipocytes and connective tissue, while other cell types, such as melanocytes, Langerhans cells, and sensory cells, are present in the epidermis and contribute to skin development and aging [4]. Among all the cells involved in skin aging, keratinocytes and fibroblasts are the most frequently studied [5].

Skin aging is a complex process influenced by intrinsic and extrinsic factors. The intrinsic factor includes genetic modulation resulting in chronological aging [6], while extrinsic factors such as ultraviolet (UV) radiation, smoking, and air pollution lead to skin photoaging [7]. Skin aging is a gradual process characterized by wrinkle formation, laxity, and fragility [6]. Histologically, photoaging and chronological aging present different characteristics but share standard features of decreased epidermal thickness and cell number, altered elastic fibers, and loss of collagen [8]. Cell senescence, such as keratinocytes and fibroblasts, has been identified as pivotal in skin aging [5].

Epigenetic modifications, which refer to potentially heritable alterations in gene expression without changes in the nucleotide sequence, have been studied for decades. The most commonly studied epigenetic regulation processes include DNA methylation, histone modification, and ncRNA regulation (including microRNAs-miRNAs, long non-coding RNAs-lncRNAs, and circular RNAs-circRNAs) [9]. Epigenetic modification begins during embryonic development and persists throughout the lifespan [10]. Age-related diseases such as Hutchinson-Gilford Progeria syndrome have been shown to interact with epigenetic regulation [11]. The concept of age-related epigenetic changes occurring in various tissues, including skin, has been widely accepted [12]. The interplay between age-related epigenetic changes and environmental cues results in intrinsic and extrinsic aging [13].

In recent decades, researchers have focused on understanding the molecular mechanisms associated with skin aging [6]. This review aims to provide a comprehensive overview of epigenetic regulation in skin aging, focusing on DNA methylation, histone modifications, and non-coding RNAs (miRNAs, lncRNAs, and circRNAs). Studies published between 2000 and 2025 were included to capture both foundational discoveries and recent advances in the field. By synthesizing clinical and experimental evidence across these years, we highlight the molecular mechanisms underlying skin aging and potential therapeutic avenues for intervention.

## 2 The correlation between skin aging and DNA methylation

Out of all the epigenetic modifications, DNA methylation is the most extensively researched. This process involves the transfer of a methyl group to the C5 position of cytosine to form 5-methylcytosine in the mammalian genome [14]. While cytosine DNA methylation is a stable epigenetic marker, it is also a highly dynamic modification that plays essential roles in various biological processes [15]. Only around 4 % of total cytosines can be modified by methylation [16]. DNA methyltransferases (DNMTs), including the *de novo* methyltransferase DNMT3 family and the maintenance methyltransferase DNMT1, are responsible for forming 5-methylcytosine from cytosine in mammals [17], 18]. The subsequent conversion of 5-methylcytosine to 5-hydroxymethylcytosine (5-hmC) by the ten-11 translocation (TETs) family, which adds a hydroxyl group, has been extensively studied in many biological processes. Previous research has demonstrated that 5hmC undergoes age-associated modifications in the DNA of aged human fibroblasts, and TET2 decreases with skin aging [19]. Differential hydroxymethylation regions of the genome with age (DHMRs) may be markers of skin aging [20]. DNMT1 regulates the natural aging of skin epidermal cells in the induced HaCaT cellular aging model [21]. DNMT1 expression decreases in senescent fibroblasts compared to younger fibroblasts, and silencing of DNMT1 in young fibroblasts induces a senescence phenotype [22]. Furthermore, DNMT1 expression increases in human skin fibroblasts (HDFs) when exposed to ultraviolet light [23]. In UV-induced skin aging, DNMT1 participates in modulating matrix proteins such as type I procollagen and the tissue inhibitor of metalloproteinases [24]. Another study indicated that DNMT1, through silencing the expression of tissue inhibitor of metalloproteinase 2(TIMP2), modulates DNA hypermethylation in UV-induced skin aging [25]. Besides UVB, UVA-induced HDFs senescence showed decreased DNMT1 expression, leading to a senescence-associated protein p53, decreased by ROS-induced down-regulation of ZEB1 [26]. A previous study confirmed that the inhibition of miR-377 reduces human dermal fibroblast senescence by targeting DNMT1. Moreover, DNMT1 reverses miR-377-mediated changes in p53 expression in HDFs. These findings illustrate that the miR-377-DNMT1-p53 axis in HSF senescence may be a novel epigenetic mechanism in skin aging [27]. These findings suggest that inhibiting DNMT1 may be a new approach to skin anti-aging strategies. There is less research on DNMT3; however, it has been reported that DNA methyltransferase Dnmt3a and Dnmt3b significantly decrease with skin aging [19]. In another study, chromatin immunoprecipitation was used to test skin fibroblasts from young and elderly individuals, and a higher level of DNMT3A protein was found to bind to the Lysyl oxidase-like 1 (LOXL1) promoter in older fibroblasts, leading to decreased LOXL1 mRNA and increased LOXL1 promoter methylation [28].

DNA methylation is a key epigenetic modification that occurs most frequently in mammals at CpG dinucleotides [15]. CpG islands are DNA sequences of GC-rich (200–2000 bp in length) with a high density of CpG dinucleotides [15]. Most CpG islands (CGIs) located at transcription start sites near gene promoters are not methylated, indicating an association with promoter activity and gene expression levels [29]. The high specificity of CpG dinucleotides ensures the heritability of methylation patterns [30]. Global DNA methylation levels decrease with age, suggesting that DNA methylation may serve as an epigenetic marker of skin aging [31]. The regulation of DNA methylation occurs primarily in a CpG context, primarily affecting cytosines [32]. Age-related hypermethylation has been shown to occur at high CpG density promoters using Illumina 450K arrays [33]. These arrays have been widely used to analyze methylation in the human genome at more than 450,000 cytosine residues.

Bormann et al. used 450K methylation arrays to investigate epidermal methylation patterns of 108 donors and concluded that age-related DNA methylation is characterized by reduced variation and increased heterogeneity of global methylation [16]. The study indicated that DNA was more methylated at CpG islands in epidermal samples from older patients. Two other studies have also demonstrated age-associated hypermethylation at high CpG density promoters [34], 35], and another study showed that hypermethylation of the methylome is observed in skin aging [36]. Whole-genome bisulfite sequencing has been used to identify age-related methylation changes at a single-base resolution, and the results show no global aberrations but localized methylation changes in promoter and enhancer regions [37]. Genome-wide DNA methylation analysis indicates that hypermethylation and hypomethylation occur in gene promoters [19], 37]. However, changes in promoter epigenetics may not represent the global regulation of gene expression. Thus, another study has demonstrated that DNA methylation changes largely with age, manifesting as blocks of the genome that are hypomethylated in older, sun-exposed epidermal samples [38]. All these age-related changes in DNA methylation were found to maintain but did not alter gene expression patterns [39]. These studies also supported the idea that global DNA methylation decreases with age, gradually losing maintenance, and resulting in increased interindividual variability [9].

**Figure 1:**
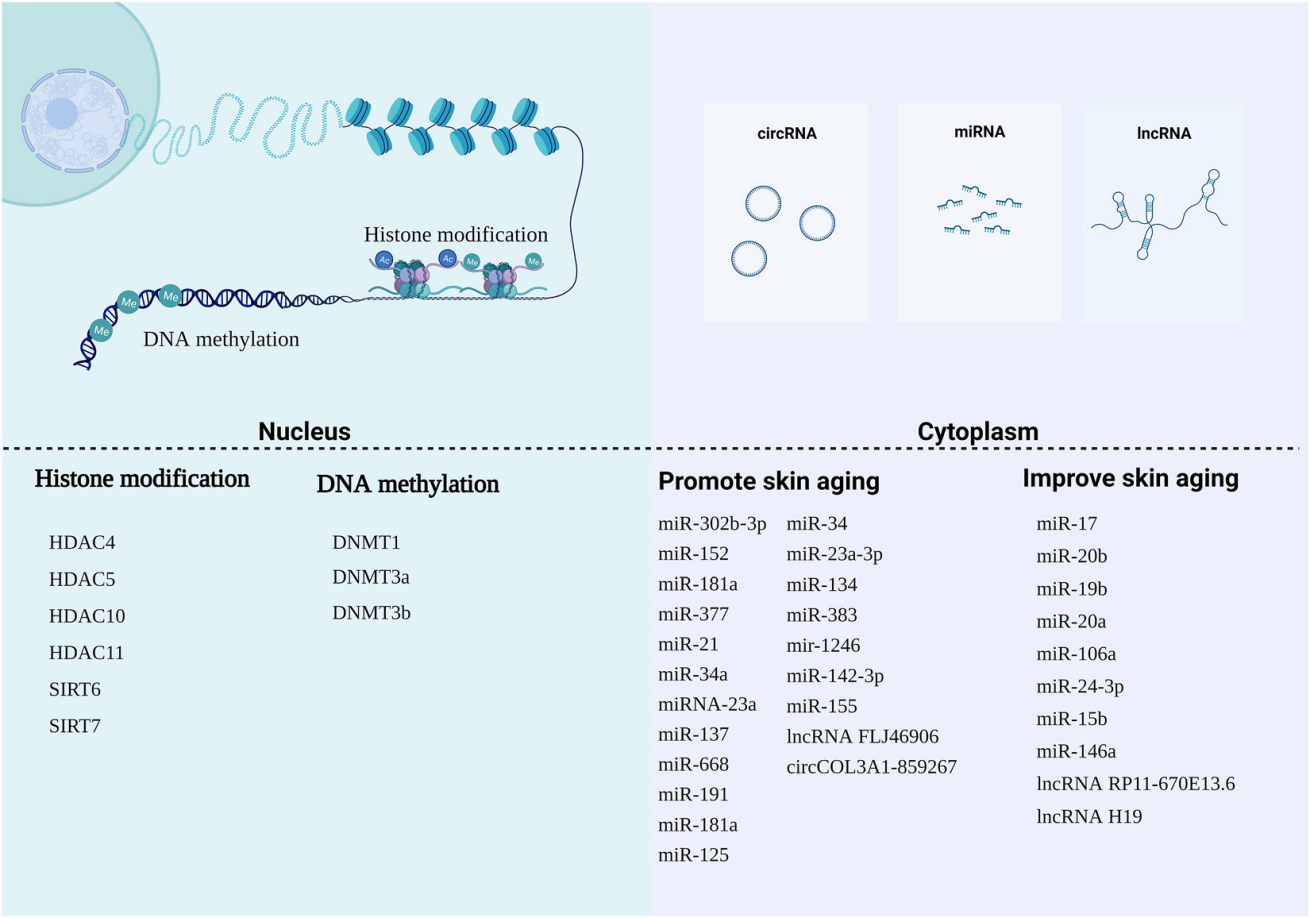
Potential mechanisms of epigenetics in skin aging. Epigenetics is crucial in understanding the underlying mechanisms of skin aging. Epigenetic modifications, such as DNA methylation, histone modifications, and non-coding RNA (ncRNA) regulation, play a significant role in this process. Within the cell nucleus, the nucleosome acts as the functional unit of chromatin, composed of DNA and histones. Histone modifications, including methylation (me) and acetylation (ac), can regulate transcription processes by influencing chromatin accessibility. DNA methylation specifically involves the transfer of a methyl group to the C5 position of cytosine in CpG islands. NcRNAs provide an additional layer of epigenetic control in the cytoplasm by regulating mRNA transcription, RNA processing, and translation. MicroRNAs (miRNAs), long non-coding RNAs (lncRNAs), and circular RNAs (circRNAs) are the major types of ncRNAs involved in skin aging. These ncRNAs play crucial roles in modulating cellular pathways and gene expression levels, contributing to the regulation of skin aging processes.

A previous study has demonstrated that monozygotic (MZ) twins exhibit epigenetic divergence in DNA methylation that increases with age and differences in lifestyle [40]. Therefore, DNA methylation in normal cells is not accurately maintained over cell divisions but is gradually modified with age. This phenomenon is referred to as “epigenetic drift” [41]. Epigenetic drift can be affected by intrinsic and extrinsic factors, with intrinsic epigenetic drift depending on genetic factors and resulting in variable outcomes [42]. Epigenetic drift manifests as, but is not limited to, the loss of DNA methylation, resulting in altered gene expression and phenotype changes [29]. Hypermethylation of CpG islands is one of the critical features of epigenetic drift [43]. However, more research is needed to explore the epigenetic mechanisms of age-related changes. Epigenetic drift has been commonly accepted as one of the markers for skin aging [29], 44] (Table 1 and Figure 1).

**Table 1:** Types of DNA Methylation related to skin aging.

Type	Description	Research models	Functions/Effects	References
5-Methylcytosine (5 mC)	Methylation of cytosine at the 5th carbon position by DNMTs	Mammalian cells, HaCaT keratinocytes, HDFs	Gene silencing, transcription repression, skin aging	[14], [17], [18], [23]
5-Hydroxymethylcytosine (5hmC)	Hydroxylation of 5 mC by TET enzymes	Aged fibroblasts, TET knockout models	Epigenetic regulation, oxidative stress response, age-related changes	[19], [20], [37]
CpG island methylation	Methylation in GC-rich regions near transcription start sites	450K methylation array, bisulfite sequencing	Regulation of gene expression, promoter silencing	[15], [29], [33]
Age-related hypermethylation	Increased methylation at CpG-rich promoters with age	Genome-wide bisulfite sequencing	Marker of aging, altered gene regulation	[31], [34], [35]
Epigenetic drift	Gradual loss or gain of methylation with age, influenced by lifestyle	Monozygotic twin studies	Interindividual variability, altered gene expression	[40], [41], [42]
UV-induced methylation	Changes in methylation patterns due to UV exposure	Human dermal fibroblasts (HDFs)	Modulation of matrix proteins (e.g., procollagen, TIMP2)	[24], [25], [26]
DNMT1-mediated methylation	Maintenance of methylation patterns during cell division	HaCaT cell aging model, UV-exposed HDFs	Involvement in senescence, anti-aging strategies	[21], [22], [27]
DNMT3-mediated methylation	*De novo* methylation established by DNMT3A and DNMT3B enzymes	Chromatin immunoprecipitation (ChIP)	Age-related decrease, regulation of LOXL1 expression	[19], [28]

Although significant progress has been made in understanding the relationship between DNA methylation and skin aging in recent years, most existing evidence remains at the level of correlation rather than causation. Current studies largely rely on *in vitro* models using cultured fibroblasts or keratinocytes, or on a limited number of human skin samples, which may not fully capture the *in vivo* dynamics of aging across different skin layers. Moreover, discrepancies exist among studies reporting age-related global DNA hypermethylation or hypomethylation trends. Such inconsistencies may partly stem from differences in detection platforms (e.g., Illumina 450K arrays vs. whole-genome bisulfite sequencing) and varying degrees of sun exposure among samples. While DNA methyltransferases such as DNMT1 and DNMT3 are thought to participate in age-associated methylation remodeling, their specific downstream targets and causal contributions to functional manifestations of skin aging (such as collagen degradation and loss of elasticity) remain inadequately defined.

Notably, age-associated “epigenetic drift” has been proposed as a hallmark of cellular aging; however, it is still unclear whether these stochastic methylation alterations drive the aging process or merely reflect accumulated environmental stress. Future research should integrate methylome, transcriptome, and proteome data – particularly at the single-cell level – to elucidate how changes in DNA methylation translate into phenotypic outcomes. Furthermore, distinguishing methylation alterations induced by intrinsic aging from those driven by extrinsic factors such as ultraviolet radiation and oxidative stress will be crucial for establishing DNA methylation as a reliable biomarker and potential therapeutic target in skin aging.

## 3 Skin aging and histone acetylation

Histone modifications, including methylation, acetylation, ubiquitination, and phosphorylation, play essential roles in skin aging [45]. Histone acetylation is a dynamic process that is controlled by histone acetyltransferases (HATs) and histone deacetylases (HDACs) [46]. These epigenetic modifications play essential roles in skin aging [47]. Previous studies have shown age-related changes in histone H4 acetylation, suggesting a decrease in acetylation over time [48]. Recent studies investigate histone acetylation using ultraviolet irradiation to induce skin photoaging. Recent studies have explored the effects of ultraviolet (UV) irritation on histone acetylation and skin photoaging. It has been shown that both chronic sun exposure and acute UV irradiation can increase the overall histone H3 acetylation level, and hyperacetylation of histone H3 may play a key role in the pathogenesis of skin photoaging [49], 50]. Additionally, the acetylation level of histone H3 (acetyl-H3) was found to increase in HDFs following UV irradiation [51]. However, exogenous pyruvate and CAPE have been shown to repair DNA damage and inhibit UV-induced acetylation of histone H3, respectively [52], 53].

Histone deacetylases (HDACs) are enzymes that remove acetyl groups from histones and other proteins. The Sirtuins family, including SIRT1-7, is one classification of HDACs [54]. Studies have shown that HDACs, especially HDAC, play a critical role in epigenetic regulation [46], 55]. UV irradiation has been shown to reduce the activity of HDACs, including HDAC4, HDAC5, HDAC10, HDAC11, SIRT6, and SIRT7, in both UV-irradiated and intrinsically aged skin. Specifically, the down-regulation of HDAC2 or HDAC7 with protein deacetylase inhibitors (HDACi) can induce skin fibroblast senescence, while the ectopic re-expression of HDAC7 can prolong the proliferative life [56]. However, there was no significant difference in the expression of HDACs in photoaged and intrinsically aged skin, suggesting that they may play a general role in increased histone acetylation associated with skin aging [56]. SIRT1 has been shown to regulate a spectrum of cellular pathways, indicating its beneficial effect on skin aging [57]. Down-regulated SIRT1 has been observed in UV and H2O2-induced skin aging, which participates in the oxidative stress response and apoptosis. Resveratrol, an activator of SIRT1, can prevent apoptosis induced by UV irradiation and H2O2 [58] (Table 2 and Figure 1).

**Table 2:** Types of Histone Acetylation related to skin aging.

Type	Description	Models	Functions/Effects	References
H3 acetylation (Ac-H3)	Acetylation of lysine residues on histone H3	UV-irradiated HDFs, skin photoaging models	Increases with UV exposure, contributes to photoaging	[49], [50], [51]
H4 acetylation (Ac-H4)	Acetylation of lysine residues on histone H4	Intrinsic aging models	Decreases with aging, linked to chromatin relaxation	[48]
SIRT1-mediated Deacetylation	Deacetylation by SIRT1 (a class III HDAC)	UV/H_2_O_2_-exposed fibroblasts, resveratrol studies	Regulates oxidative stress response, prevents apoptosis	[54], [57], [58]
HDAC7-mediated regulation	Deacetylation by HDAC7, involved in maintaining proliferative potential	Aged fibroblasts, HDAC inhibitors (HDACi)	Loss of HDAC7 induces senescence; re-expression extends proliferation	[56]
UV-induced HDAC inhibition	UV exposure reduces HDAC activity, including HDAC4, HDAC5, HDAC11	UV-irradiated fibroblasts	Decreased HDAC activity correlates with photoaging	[55], [56]
SIRT6 and SIRT7 in aging	Deacetylation by SIRT6 and SIRT7, involved in cellular aging processes	Intrinsically aged skin, UV exposure models	Down-regulated with aging, regulates DNA repair	[54], [56]
CAPE and pyruvate Treatment	CAPE and pyruvate reverse UV-induced acetylation	HDFs treated with CAPE/pyruvate	Repair DNA damage, inhibit histone acetylation	[52], [53]

Although numerous studies have explored histone acetylation in skin aging, several limitations remain. Most investigations focus on H3 and H4 acetylation, with other post-translational modifications such as methylation, ubiquitination, and phosphorylation largely underexplored. Additionally, many studies rely on *in vitro* models or UV-induced photoaging, which may not fully recapitulate the complex interactions and heterogeneity of skin aging *in vivo*. The reported effects of HDACs and sirtuins on aging phenotypes are sometimes inconsistent, potentially due to differences in experimental conditions, cell types, or UV dosages. Moreover, while interventions such as exogenous pyruvate or resveratrol show promise in modulating histone acetylation, their translational relevance remains to be validated in clinical studies. Future research should integrate multi-omics approaches, examine histone modifications across diverse skin cell types, and clarify causal links between specific acetylation patterns and functional aging phenotypes, thereby providing more robust therapeutic targets for delaying skin aging.

### 3.1 Skin aging and microRNA

#### 3.1.1 MicroRNA in cellular senescence

Senescent cells, including fibroblasts and keratinocytes, accumulate in various tissues over time and contribute to tissue dysfunction and aging-associated phenotypes. Fibroblast senescence can secrete the senescence-associated secretory phenotype, contributing to skin aging [8]. Keratinocyte senescence plays a crucial role in epidermal time-related changes. The senescence of both keratinocytes and fibroblasts leads to loss of integrity and dysfunction of the skin.

MicroRNAs (miRNAs or miRs) are small non-coding RNAs that regulate post-transcriptional gene expression by degradation of target messenger RNA or transcriptional inhibition. The development of skin and hair is governed by complex programs of gene activation and repression, as well as microRNA-dependent regulation of gene expression, which together maintain normal skin and hair follicle development, homeostasis, and cycling. For example, miR-148a plays a critical role in skin homeostasis and hair follicle cycling by regulating Rock1 and Elf5; its loss accelerates the anagen phase and alters stem cell activity, highlighting its role as an important epigenetic regulator in maintaining tissue homeostasis and regeneration. miRNAs also act as controllers of protein expression, modulating multiple functions such as aging-associated proliferation and differentiation, inflammation, and cell death [59]. Increasing evidence has demonstrated that senescence-associated microRNAs regulate molecular mechanisms in the skin aging process [2]. Although the role of miRNA in skin aging has not been extensively studied, it may play an important role in regulating the skin aging process of the epidermis and dermis, especially in cellular senescence, decreased collagen synthesis, and barrier dysfunction. A previous study indicated increased expression of miR‐302 b‐3p in senescent fibroblasts and aged skin tissues. Inhibition of miR-302 b-3p can delay skin fibroblast senescence and increase the expression of SIRT1. Conversely, overexpression of miR-302 b-3p contributes to skin aging by targeting c‐Jun N‐terminal kinase 2(JNK2) [60]. Furthermore, researchers found that the expression of miR-152 and miR-181a increased in human dermal fibroblast senescence [61]. However, some other miRNAs, such as miR-17 and miR-20 b, are known to be downregulated during human dermal fibroblast senescence and skin aging in humans [62]. Three miRNAs (miR-92a, miR-15 b, and miR-125a-3p) were downregulated in dermal skin fibroblasts [63]. In the context of skin aging, various miRNAs are dysregulated in human dermal fibroblasts and epidermal keratinocytes. Specifically, miR-17, miR-19 b, miR-20a, and miR-106a were shown to be downregulated in a replicative cell aging model of skin fibroblasts [64]. Conversely, miR-377 expression was found to be significantly higher in passage-aged human skin fibroblasts, and its inhibition has been suggested to reduce cellular senescence by targeting DNMT1 in the miR-377-DNMT1-p53 axis [27]. Furthermore, miR-21 was found to be upregulated in epidermal keratinocytes during skin aging and may contribute to chromatin remodelling and epidermal differentiation. It was also found to increase the susceptibility of age-related changes by regulating special-A T-rich-sequence-binding protein-1(Satb1) in epidermal keratinocytes [65]. In another study, Kruppel-like factor 4 (KLF4), a transcription factor, was found to decrease during keratinocyte replicative senescence. Senescence-associated miR-34a was shown to regulate post-transcriptionally Klf4 expression, and the silencing of Klf4 induced keratinocyte senescent phenotype and overexpression of miR-34a [66]. Additionally, miR-30a was found to impact keratinocyte apoptosis and skin barrier function in an organotypic culture model by regulating several gene targets, including LOX, IDH1, and AVEN, in keratinocytes [67]. Lamin1 has been found to decrease in cells from progeria patients [68], which intrigued researchers to test its same tendency in senescent human dermal fibroblasts, keratinocytes, and aged skin tissue. The reduction of Lamin B1 is mediated by miRNA-23a, which inhibits LMNB1 messenger ribonucleic acid (RNA) translation and reduces transcription [69] (Table 3 and Figure 1).

**Table 3:** Summary of MicroRNAs in skin aging.

Type	Description	Models	Functions/Effects	References
miR-302b-3p	Upregulated in senescent fibroblasts and aged skin	Senescent fibroblasts	Promotes aging by targeting JNK2; inhibition delays senescence and increases SIRT1 expression	[60]
miR-152, miR-181a	Upregulated in fibroblast senescence	Human dermal fibroblasts	Regulates collagen XVI and integrin α5, affecting ECM and senescence	[61]
miR-17, miR-20b	Downregulated during senescence	Human dermal fibroblasts	Associated with decreased collagen synthesis and cell senescence	[62]
miR-92a, miR-15b, miR-125a-3p	Downregulated in dermal fibroblasts	Senescent fibroblasts	Associated with skin barrier dysfunction and collagen degradation	[63]
miR-377	Upregulated in passage-aged fibroblasts	Human dermal fibroblasts	Promotes senescence by targeting DNMT1 in the miR-377-DNMT1-p53 axis	[27]
miR-21	Upregulated in keratinocytes during aging	Epidermal keratinocytes	Affects chromatin remodeling, differentiation, and Satb1 regulation	[65]
miR-34a	Regulates Klf4 expression post-transcriptionally	Keratinocyte senescence model	Promotes senescence phenotype by targeting KLF4	[66]
miR-30a	Involved in skin barrier function	Keratinocyte culture model	Regulates apoptosis and key genes like LOX, IDH1, and AVEN	[67]
miR-23a	Inhibits LMNB1 translation	Dermal fibroblasts, keratinocytes, aged skin	Reduces Lamin B1 levels, contributing to skin aging	[69]
miR-137, miR-668	Upregulated in senescent fibroblasts	Fibroblast senescence models	Linked to p16INK4A and p53 markers, promoting senescence	[73]
miR-191	Prevents G1-S phase transition	Fibroblast senescence	Facilitates cell cycle arrest and senescence	[2]
miR-24-3p	Overexpressed during irradiation	Skin irradiation model	Protects collagen synthesis and skin barrier by targeting p38	[76]
miR-34	Alters ECM components	Human dermal fibroblasts	Modulates MMP-1, COL1A1, and elastin expression	[77]
miR-1246	Promotes UVB-induced apoptosis	UVB-irradiated keratinocytes	Inhibits rhotekin 2, contributing to skin damage	[85]
miR-142-3p	Upregulated in UVB-induced DNA damage	Human keratinocytes	Promotes DNA damage response	[86]
miR-155	Decreased under UV exposure	UVA-irradiated dermal fibroblasts	Upregulates c-Jun, affecting collagen expression	[89]
miR-146a	Anti-aging miRNA	Human dermal fibroblasts	Inhibits P21WAF-1, p16, and p53 by targeting Smad4, preventing senescence	[90]

Although increasing evidence supports the role of microRNAs in regulating cellular senescence and skin aging, several limitations remain. Most studies focus on a limited set of miRNAs and primarily use *in vitro* fibroblast or keratinocyte models, which may not fully capture the complex cellular interactions and microenvironment of aging skin *in vivo*. Moreover, different studies report contradictory expression trends for some miRNAs (e.g., miR-17, miR-20 b, miR-377), possibly due to differences in experimental conditions, donor age, or tissue sources. The functional consequences of miRNA dysregulation in dermal versus epidermal layers, as well as their interactions with other epigenetic mechanisms such as DNA methylation and histone modifications, remain incompletely understood. Future research should integrate multi-omics data, including transcriptomic, epigenomic, and proteomic analyses, preferably at the single-cell level, to clarify how miRNA-mediated regulation contributes causally to skin aging phenotypes. Additionally, distinguishing miRNA changes induced by intrinsic aging from those caused by extrinsic factors, such as UV exposure or oxidative stress, is crucial for developing reliable therapeutic targets for delaying skin aging.

### 3.2 MicroRNA in collagen synthesis

Apart from cellular senescence, skin aging is also characterized by a decrease in the production of extracellular matrix proteins such as type I and III collagens due to the activation of matrix metalloproteinases (MMPs) [70], 71]. Replicative senescence is characterized by enhanced senescence-associated *β*-galactosidase activity and increased expression of key mediators such as p53, p16INK4a, and p19Arf [72]. Reduced collagen and elastin are common signs of skin aging, resulting from altered gene expression that leads to the senescent phenotype. Fibroblast senescence contributes to skin aging by secreting a senescence-associated secretory phenotype that reduces proliferation by impairing the release of essential growth factors and enhancing the degradation of the extracellular matrix via MMPs activation [8]. The ARF/p53 and p16INK4A/RB pathways and *β*-galactosidase activity are correlated with upregulated levels of miR-137 and miR-668 as well as p16INK4A and p53 senescence markers [73]. MiR-191 can also prevent the G1-S phase transition, resulting in cell cycle arrest and a quiescent state, which facilitates senescence processes [2]. Upregulated miR-181a directly inhibits the expression of collagen XVI, a minor component of the cutaneous ECM, in senescent fibroblasts [74]. miR-125 inhibits the expression of type I collagen protein, the primary cause of dermal skin aging [75]. Overexpression of miR-24–3p inhibits the effect of irradiation on the skin barrier and collagen synthesis by targeting p38 [76]. MiR-34 in HDFs alters MMP-1, COL1A1, and elastin expression and cell function, indicating that miRNAs can significantly affect how quickly the dermis ages [77]. Overexpression of miR-23a-3p leads to cellular senescence by targeting hyaluronan synthase 2, while overexpression of miR-152 and miR-181a regulates integrin α5 and collagen XVI levels, resulting in changes in the dermis and epidermis and contributing to cellular senescence during skin aging [61] (Table 3 and Figure 1).While miRNAs clearly influence collagen synthesis and ECM remodeling, most studies rely on *in vitro* fibroblast models, which may not fully reflect *in vivo* skin aging. Effects of miRNAs can be context-dependent and sometimes contradictory, and their interactions with MMPs and other epigenetic mechanisms remain unclear. Future studies using integrated multi-omics and single-cell approaches are needed to clarify causal roles and distinguish intrinsic versus extrinsic aging effects.

### 3.3 MicroRNA in skin photoaging

Solar UV radiation, an important environmental carcinogen, can be divided into three regions based on wavelength: short-wave UVC, mid-wave UVB, and long-wave UVA [78]. Only the longer wavelengths can penetrate the dermis. Exposure to UV light is a dominant factor that contributes to skin aging, with ultraviolet B (UVB) radiation inducing DNA damage, oxidative stress, and inflammation, affecting the immune system and contributing to skin aging [79]. In a skin model of mice aged by UVB irradiation, the expression of miRNAs was significantly altered [80]. Previous studies have shown changes in miRNA expression upon UV irradiation, including increased expression of miR-27a, miR-145, miR-383, miR-34a, miR-134, and miR-1246, and decreased expression of miR-155, miR-663 b, miR-3648, and miR-6879. MiR-27a reduces cell apoptosis by facilitating the removal of cyclobutane pyrimidine dimers [81]. Among them, miR-383, miR-34a, and miR-134 were differentially expressed in a study identifying microRNA in chronological and photoaging human skin [82]. In UVB-irradiated keratinocytes, 44 miRNAs were upregulated or downregulated in a time-dependent manner [83], and the expression of 55 miRNAs changed under chronic sun exposure-induced skin aging [82]. MiR-34a, miR-134, and miR-383 target the p53 complex via G1 phase arrest and the cell cycle to accelerate aging [82]. Under UVB-induced skin aging, miR-34a deteriorates collagen structure by activating matrix metalloproteinases, eventually decreasing skin elasticity and causing wrinkle formation [84]. Some miRNAs, such as miR-1246, promote skin aging by upregulating UVB-induced apoptosis by inhibiting the expression of rhotekin 2 [85], and miR-142–3p is upregulated in UVB-induced DNA damage in human keratinocytes [86]. However, other microRNAs, such as miR-15 b, decreased in photoaged skin by targeting SIRT4 and interacting with senescence-associated mitochondrial dysfunction [87]. The downregulation of miR-101 was insufficient to block the senescence phenotype of UVB-induced human fibroblasts [88]. Under UVA irritation, miR-155 significantly decreased, resulting in c-Jun upregulation and affecting the collagen gene in human dermal fibroblasts [89]. However, miR-146a has been demonstrated to have an anti-aging effect by inhibiting senescence-associated genes such as P21WAF-1, p16, and p53 by targeting Smad4 [90] (Table 3 and Figure 1).Although many studies have identified UV-induced miRNA changes in skin photoaging, most rely on *in vitro* keratinocyte or fibroblast models or limited animal studies, which may not fully capture the complex *in vivo* environment. Some miRNAs show contradictory effects depending on cell type, UV dose, or exposure duration. Moreover, the causal mechanisms linking specific miRNAs to functional photoaging outcomes, such as collagen degradation and wrinkle formation, remain incompletely understood. Integrating miRNA profiling with transcriptomic, proteomic, and single-cell analyses in human skin will be essential to clarify their precise roles and therapeutic potential.

### 3.4 Skin aging and Lnc RNA

Previous studies have shown that the expression of long non-coding RNA (lncRNA) changes in human dermal fibroblasts under ultraviolet irradiation. One study detected 34 differentially expressed microRNAs through high-throughput sequencing in UVA-irradiated human skin fibroblasts (HDFs) at 10 J/cm2 and found that the miRNA-lncRNA-mRNA network played a crucial role in skin photoaging, as demonstrated by functional annotation analysis and pathway enrichment using Gene Ontology and KEGG [91]. Another study found that the lncRNA FLJ46906 is upregulated with aging in skin fibroblasts both *in vitro* and *in vivo*, modulating aging-related genes including IL1B, IL6, and CXCL8, as well as TGFB1 and ELN through NF-κB and AP-1 [92]. Similarly, another study found that lncRNA RP11-670E13.6 binds to miR-663a in UVB-exposed primary human skin fibroblasts and regulates the disinhibition of Cdk4 and Cdk6, thereby alleviating UV-induced cell senescence [93]. However, a different study has shown that the lncRNA H19 is down-regulated in aging dermal fibroblasts. This lncRNA H19 upregulates IGF2 by targeting the protein of miR-296–5p, activating the PI3K/mTOR pathway, and upregulating the expression of AQP3 in HDFs by binding to miR-296–5p [94]. Therefore, the upregulation of LncRNA H19 may promote HDFs activity and inhibit skin aging. In keratinocytes, UVB-induced damage resulted in decreased expression of the non-coding RNA nc886, leading to increased expression of matrix metalloproteinase-9 (MMP-9), type IV collagenase, and cyclooxygenase (COX-2), ultimately accelerating skin aging [95]. Currently, existing research focuses on the role of non-coding RNA in UV-induced cellular senescence and skin aging, providing new insights into potential therapeutic targets induced by ultraviolet radiation. However, more research is needed to support the regulatory network of more non-coding RNAs in skin aging (Table 4 and Figure 1).Although lncRNAs have been implicated in skin aging, their functional roles remain inconsistent across studies, reflecting the complexity of regulatory networks.Contradictory results, such as the opposing roles of lncRNA H19 in aging, highlight the complexity and context-dependence of lncRNA function. Comprehensive *in vivo* studies and integrative analyses combining lncRNA, miRNA, and mRNA networks are needed to clarify causal mechanisms and identify reliable therapeutic targets for skin aging.

**Table 4:** Summary of LncRNAs and circRNAs in Skin Aging.

Type	Name	Description	Models	Functions/Effects	Ref.
LncRNA	FLJ46906	Upregulated with aging	*In vitro* and *in vivo* fibroblast models	Modulates IL1B, IL6, CXCL8, TGFB1, and ELN via NF-κB and AP-1, contributing to skin aging	[92]
	RP11-670E13.6	Binds miR-663a to alleviate UVB-induced senescence	UVB-exposed human skin fibroblasts	Regulates Cdk4 and Cdk6 expression to alleviate UV-induced senescence	[93]
	H19	Downregulated in aged fibroblasts	Aging dermal fibroblasts	Upregulates IGF2 and activates PI3K/mTOR pathway; promotes AQP3 expression and inhibits skin aging	[94]
	nc886	Decreased under UVB damage	UVB-irradiated keratinocytes	Increases MMP-9, COX-2, and type IV collagenase, accelerating skin aging	[95]
circRNA	circCOL3A1-859267	Regulates type I collagen expression	Human dermal fibroblasts	Down-regulation contributes to UV-induced photoaging by reducing collagen synthesis	[97]
	Differential circRNAs	29 circRNAs altered in photoaged HDFs	Photoaged HDFs via high-throughput sequencing	Several circRNAs are up- or down-regulated, involved in collagen synthesis and aging	[98]
	CircRNAs in eyelid skin	571 circRNAs differentially expressed	Aged vs. young eyelid skin samples	Potential biomarkers for skin aging; role in collagen synthesis and ECM regulation	[98]
	Hsa-miR-588, hsa-miR-612, hsa-miR-4487, hsa-miR-149-5p, hsa-miR-494-5p	Participate in circRNA-miRNA networks	CircRNA interaction networks	May regulate key pathways in ECM maintenance and skin aging	[98]

### 3.5 Skin aging and circle RNA

Circular RNAs (circRNAs) have been shown to play an important regulatory role in skin photoaging, but their function in skin aging remains to be fully elucidated. Reduced type I collagen synthesis is a hallmark of skin photoaging [96]. Previous studies have found that circCOL3A1-859267 regulates the expression of type I collagen in HDFs, and down-regulation of this circRNA may play a regulatory role in UV-induced photoaging [97]. Additionally, Peng et al. identified 29 differentially expressed circRNAs (12 upregulated and 17 down-regulated) in photoaged HDFs using high-throughput sequencing. Another study analyzed the expression profile of circRNAs in four pairs of aged and non-aged eyelid skin samples and found 571 differentially expressed circRNAs, of which 348 and 223 circRNAs were differentially expressed in aged and young skin samples, respectively. These differentially expressed circRNAs may hold clinical value as biomarkers for the skin aging process. Moreover, several circRNAs, such as hsa-miR-588, hsa-miR-612, hsa-miR-4487, hsa-miR-149–5p, and hsa-miR-494–5p, may be involved in a circRNA-miRNA interaction network [98]. However, most studies are limited to expression profiling, with few functional validations. The small sample sizes and lack of *in vivo* evidence restrict their generalizability. Future research should focus on mechanistic studies to clarify how circRNAs influence cellular senescence and extracellular matrix integrity, and to evaluate their potential as therapeutic targets in skin aging(Table 4 and Figure 1).

## 4 Future perspectives

Epigenetic alterations are central drivers of skin aging, encompassing DNA methylation, histone modifications, and chromatin remodeling. While age-associated DNA methylation, characterized by reduced variability and increased heterogeneity, serves as a predictor of biological age and overall skin health, significant gaps remain in understanding the contributions of DNMT3 enzymes and RNA modifications such as *N*6-methyladenosine (m6A). Future studies should leverage both bulk and single-cell epigenomic approaches to map DNA methylation dynamics across diverse skin cell types and aging stages, providing mechanistic insight and potential therapeutic targets.

Non-coding RNAs, including miRNAs, lncRNAs, and circRNAs, are critical regulators of cellular senescence, collagen synthesis, and skin barrier integrity. Many studies to date focus on fibroblasts and keratinocytes, leaving melanocytes, Langerhans cells, vascular, and immune components underexplored. Future research should systematically examine ncRNA expression and function across these additional cell types, their interactions with gene networks, cell cycle checkpoints, apoptosis, proliferation, differentiation, and responses to UV-induced DNA damage. Furthermore, integrating ncRNA studies with innovative delivery systems, such as nanoparticles or exosomes, could bridge mechanistic insights with translational anti-aging strategies.

Histone modifications, particularly acetylation, have been extensively studied, with H4 acetylation decreasing with age and H3 acetylation increasing in response to acute and chronic UV exposure. However, other post-translational modifications, including methylation, phosphorylation, and ubiquitination, remain underexplored. Future investigations should broaden the scope to these additional modifications and examine the roles of histone deacetylases and histone-modifying enzymes in coordinating cellular senescence and extracellular matrix remodeling. Experimental interventions, such as exogenous pyruvate and ethyl caffeic acid, highlight the potential to modulate histone acetylation for photoaging mitigation, but translational validation is needed.

Collectively, future research should aim to construct an integrated, multi-layered epigenetic model of skin aging, combining DNA methylation, histone modification, and ncRNA regulation across multiple cell types and experimental models. Incorporating single-cell epigenomics, advanced delivery strategies for ncRNA-based interventions, and clinically relevant human studies will be essential to identify robust therapeutic targets, advance translational applications, and ultimately develop precise interventions to delay intrinsic and extrinsic skin aging.

## 5 Conclusions

Skin aging is orchestrated by a complex interplay of epigenetic mechanisms, including DNA methylation, histone modifications, and non-coding RNAs, which collectively regulate cellular senescence, extracellular matrix integrity, and responses to environmental stressors such as ultraviolet radiation. DNA methylation and associated enzymes, such as DNMT1, modulate collagen synthesis and matrix remodeling, while histone modifications influence chromatin accessibility and gene expression during intrinsic and photo-induced aging. Non-coding RNAs – including miRNAs, lncRNAs, and circRNAs – act as critical regulators of skin cell function, yet current studies are largely confined to fibroblasts and keratinocytes, with limited investigation in melanocytes, Langerhans cells, and vascular components.

Despite substantial advances, mechanistic insights remain fragmented, and translational applications are still limited. Future research integrating multi-layered epigenetic regulation across diverse cell types, combined with innovative delivery strategies for ncRNA-based interventions, holds promise for developing targeted anti-aging therapies. By bridging molecular mechanisms with therapeutic innovation, the field can advance toward interventions that delay skin aging, mitigate photoaging, and ultimately improve skin health and resilience.
